# Introducing Augmented Reality Technology to Enhance Learning in Pharmacy Education: A Pilot Study

**DOI:** 10.3390/pharmacy8030109

**Published:** 2020-06-30

**Authors:** Jennifer Schneider, Melanie Patfield, Hayley Croft, Saad Salem, Irene Munro

**Affiliations:** Discipline of Pharmacy, School of Biomedical Sciences & Pharmacy, University of Newcastle, Callaghan, NSW 2308, Australia; jennifer.schneider@newcastle.edu.au (J.S.); Melanie.Patfield@uon.edu.au (M.P.); Hayley.Croft@newcastle.edu.au (H.C.); Saad.Salem@newcastle.edu.au (S.S.)

**Keywords:** augmented reality, student-centred learning, medicines, pharmacy

## Abstract

There is increasing use of augmented reality (AR) technology, which combines the virtual and real world, in the tertiary education sector. AR enables flexibility in student learning, since this technology may be used in the face to face setting and may also be accessed by students at any time outside of this setting. The purpose of this study was to develop an AR tool and investigate its effectiveness for learning about the medication naloxone using AR in a MagicBook; and determine student opinions on its acceptability and usability. Using a sequential explanatory, mixed method design, 25 undergraduate pharmacy students were recruited to participate in the study. Pre- and post-tests were used to measure changes in knowledge and a survey was used to collect information on the usability and acceptability of AR for learning. The findings of the study indicated that AR technology was able to support student learning on the chosen topic, showing 42% improvement in quiz score *p* < 0.0001, and that students found using AR was stimulating, interactive, engaging and easy to follow. Thus, AR technology could be an effective way to enhance student learning about medicines.

## 1. Introduction

In the health and education sectors, the growing use of immersive technologies, such as Augmented Reality (AR) and Virtual Reality (VR), have generated new opportunities to enhance the development of clinical and communication skills of students as well as the way in which they learn [1,2]. While VR immerses the learner in a virtual world from where they cannot see the surrounding real world, AR superimposes computer-assisted virtual objects such as pictures, videos, text or animations into the real world creating a reality for the learner that is enhanced or augmented [3]. Thus AR supplements reality rather than completely replacing it [4]. It has been suggested that using AR for learning enables students to have a more absorbing and engaging environment without the loss of real world experiences [2,5].

The use of AR as a teaching resource represents a student-centred approach to learning. AR tools may be used to augment traditional teaching modalities such as face to face learning. These resources also provide students with the flexibility to easily access and revisit content and concepts at any time or location outside of the classroom setting. It is suggested that students who actively participate in an authentic learning environment focus their attention [6], not only learning more but remembering more [7,8]. Furthermore, turning a conceptual or difficult to visualise subject into a real and observable concept enables students to develop an increased understanding of the topic area [9]. AR technology increasingly supports students in their understanding of complex and abstract topics [10,11] as well as increasing the learning motivation of students [12]. AR technologies for learning have been successfully implemented in medical, nursing, and dentistry education as well as a variety of other allied health disciplines [13,14,15]. Similarly, media resource *Pharmacy Times* developed an AR application that reveals extra content when readers scan the pages [2]. However, as yet, there is limited evidence on the use of AR technology in pharmacy teaching and learning.

A key learning area for pharmacy students is the use of medicines and students must acquire comprehensive knowledge about an extensive range of medicines and devices. Students must also become well versed in the application of this knowledge so that, when practising as a pharmacist, they will be able to optimise patient care through provision of appropriate medication and device counselling. The learning and appropriate development of such skills can be enhanced when students actively participate in the learning process [16]. Tertiary programs tasked with preparing future pharmacists rely on providing pharmacy students with opportunities to develop familiarity with all aspects of medicines and their use. As part of face to face teaching in pharmacy, access to actual medication and devices is often provided to assist students in familiarising themselves with medicinal products and devices. However, for students to access products in class, a large number of many different medicines and devices must be purchased. The purchase of these products may incur considerable expense, and, in some cases, the cost of these products may preclude their purchase for use in the classroom. Some medications, such as opioids, are highly restricted in terms of access, handling and storage, and this may limit their use in classroom teaching. Medicinal products and devices also have expiry dates and changes in their product design or packaging may occur over time, requiring the purchase of replacement of the products used for teaching at regular intervals. Further to this, while students may be provided with opportunities to access medicines in one-off face to face sessions, outside of the classroom students are unable to access or review these medicines or devices. The window of opportunity to examine medicines may also be limited to only when students are enrolled in a particular subject and there may be no other times when the medicines are available for revision. For example, students may learn about a particular medication in the earlier years of their training and then, when the use of this medicine is revisited in a complex case setting in the later years, access to products and devices to revise their learning may not be available.

These limitations provided the motivation to explore the use of AR in pharmacy teaching. AR has the ability to provide visual images of products and devices for use inside and outside the classroom setting. By purchasing only one product or device, images such as 360-degree photos or videos of the product or device may be produced, while images of foil strips of tablets or other components contained within the packaging may also be captured for use in AR resources. If the presentation of a product or the design changes only one new product needs to be purchased to update the AR resource. Devices may be disassembled so students can view the various components. Using AR enables students to see products, replacing the need to purchase multiple medications and devices for use once or twice a year. For products with restricted access, photos or videos can be produced by accessing these on only one occasion. While AR may be used to produce standalone resources where a picture or other “trigger” is scanned to provide a video, picture or other information, it may also be incorporated into interactive learning modules that provide visualisations and augmented information about medicines to the student, delivered using a flexible online platform that would otherwise not be routinely available. It was this approach of developing a training module which incorporated AR elements which was evaluated in this study.

Naloxone, an opioid antagonist, was selected as the subject of this AR study. From 1 February 2016, naloxone, when used for the treatment of opioid overdose, became classified as a *Pharmacist Only* medicine, making it more widely available over the counter from Australian community pharmacies without requiring a prescription from a medical practitioner. For all other purposes, naloxone remains classified as *Prescription Only*. There are no data which indicate that there has been increased naloxone usage since its rescheduling, or whether expanding the provision of naloxone is associated with greater risk-taking by patients or any increase in drug use [17]. However, there remains a need for pharmacists and pharmacy programs to provide more specific training for this previously infrequently used medication. Medication counselling for naloxone use in overdose is complex and requires the pharmacist to explain signs and symptoms of overdose, how to assemble and use naloxone ampoules and a protocol for use in an overdose scenario. Given that requests for this medication may be infrequent, the development of a training module which could be re-accessed to refresh knowledge from time to time would also be useful. Thus the aim of this study was to develop an AR learning tool focusing on competencies required for naloxone supply in community pharmacies, to include in an undergraduate Bachelor of.Pharmacy (Honours) program, and to evaluate its acceptability, usability and effectiveness for student learning.

This study was approved by the Human Research Ethics Committee at the University of Newcastle, Australia, approval number, H-2013-0151.

## 2. Materials and Methods

This study was conducted between March and October 2018.

### 2.1. Selecting the Appropriate AR Software

Before commencing the design and application research phase, three AR systems were evaluated to determine their suitability for use in this study. Feasibility was established using the following principles: affordability, user friendliness, design and availability. Initially, a web-based user portal was identified as an efficient and cost-effective method of creating an AR application. This system has the ability to create an AR experience without prior knowledge of programming code [18]. Three web marker-based AR systems were identified as a potential technological base of this study: Wikitude (https://www.wikitude.com/), HP Reveal^®^ and Blippar (https://www.blippar.com/). The advantages of these options are summarised in Table 1.

Wikitude^®^ is a popular AR system which provides a robust platform for AR production and that produces web-based AR content without coding knowledge. Content produced is stored on the cloud [19]. While there is a limited trial version which is free, cost of access to the level required for this study was not affordable and, while the newer version of Wikitude^®^ was able to perform many sophisticated tasks, in terms of ease of use for creating content required for this study, it was more difficult to use than the other options.

HP Reveal^®^ provided an easily accessible web-based AR platform that linked to a cloud database. Access to the platform to create AR resources was free. Once created, AR trigger images, videos, audio or 3D models, which are immediately available and can be visualised using the camera of any mobile device [20]. The time required to learn how to create an AR resource was short, with the process being simple and straightforward. A limitation of the HP Reveal^®^ software was that all videos had to be under 100 MB in size [20].

Blippar^®^ is a user-friendly web-based AR browser that enables the user to interact with tangible objects through the camera or touchscreen of a mobile device [20]. As with HP Reveal^®^, it can visualise images, audio, video and 3D models over the trigger images, but there is a cost involved [20], which makes it less affordable.

### 2.2. Development of the AR Tool

Overall, HP Reveal^®^ appeared to be the most suitable for this study and was chosen as the platform to develop an AR MagicBook to build student learning about the medication supply process for naloxone.

#### 2.2.1. Development of Therapeutic Content

Three currently practicing and academic pharmacists were individually consulted to determine the learning objectives for the AR activity and the therapeutic content to include within the AR MagicBook, as presented in Table 2.

#### 2.2.2. Development of the AR MagicBook

**Figure 1:**
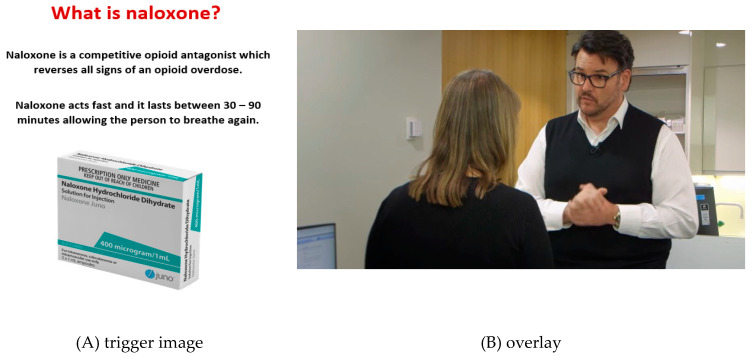
Sample page from the AR MagicBook on naloxone showing the box of medication (**A**) and, screen capture of the video played (**B**) when the trigger image is scanned.

A MagicBook is like a traditional book in appearance; pages can be turned, pictures looked at and the text read without any additional technology. However, each page also contains an AR image relevant to the content [21]. Known as trigger images, for this MagicBook they were created from photographs of medication boxes, scripts, videos and cartoons, saved as jpeg files and uploaded on to the web portal of HP Reveal^®^. The trigger image could then be used to conceal a linked item known as an overlay which provides more information, for example the medications inside the box or a video of a pharmacist providing over the counter information on the use of the medication to a customer. An example page can be seen in Figure 1. The trigger image was the naloxone medication box with an overlay item of a customer requesting Naloxone and a pharmacist supplying the medicine in a simulated patient interaction which is hidden from view. An option is to include two or more overlays which are sequentially revealed with AR markers. The built in AR markers provide attentional guidance that leads students to view 3D models and videos that are seen as real [22]. When a tablet or iPhone, with HP Reveal^®^ downloaded, is held over the image of the medication box it triggers the appearance of the overlay.

Through an iterative process of consultation, the final AR images for including in the 14-page book were selected with appropriate information included with the images. The MagicBook was made available to students as a hard copy version for first time use of the module. Subsequently it was made available online for students to access at any time and in any place through the learning management system Blackboard.

### 2.3. Experimental Design

The AR MagicBook was initially used with undergraduate students in the Bachelor of Pharmacy (Honours) degree at the University of Newcastle, Australia. A convenience sampling method was used and students from all four years of the Bachelor of Pharmacy (Honours) degree were invited to participate in the study which was conducted during a separate session outside of student’s usual course requirements, in their own time. Students who consented to participate individually completed the AR activity on a single occasion by attending a session which included a pharmacy academic who was able to provide clinical context and a researcher who managed the completion of the AR research activity sequentially as outlined below. A sequential explanatory, mixed method design (qualitative survey data and quantitative analysis of test data) was used.

Students were asked to complete a demographic survey followed by a pre-test to collect baseline knowledge of the topic (Step 1). Students were then provided with a tablet or iPad on to which HP Reveal^®^ had been downloaded and were shown how to proceed with the module. Working with a copy of the MagicBook, one page at a time, the students held the tablet over the trigger image to reveal the overlay(s). Guided by the AR markers, students worked at their own pace so that when they had worked through the information presented, they moved on to the page(s) that followed (Step 2). Students then completed a post-test, which included the same questions as the pre-test. Lastly, students were asked to complete a survey to collect their opinions on the usability and acceptability of the AR module for learning (Step 3).

The questions contained in the pre- and post-tests were identical to determine whether participants had acquired content knowledge after completing the AR MagicBook activity. The pre- and post-tests contained four multiple choice questions, each with four optional answers, and one short-answer question to assess the students’ knowledge of the process of naloxone supply (Table 3).

### 2.4. Statistical Analysis

Student results from the pre- and post-tests were analysed using SPSS (IBM Corp. Released 2017. IBM SPSS Statistics for Windows, Version 25.0. Armonk, NY, USA: IBM Corp). The total of correct responses for each question pre- and post-test were calculated, and two-tailed paired *t*-tests were conducted to determine significant differences for each question as well as for the overall results.

Student acceptance of using the MagicBook for learning was measured with a usability/acceptability survey comprising six statements using a five-point Likert scale, with scoring from 1 (strongly disagree) to 5 (strongly agree). The survey also included two open-ended questions for collection of qualitative information from the students on what they liked about using AR and possible improvements for future medicines learning. Thematic analysis was used to group and analyse the responses

## 3. Results

### 3.1. Demographic Data

A total of 25 students completed the AR module, 14 females and 11 males. Most of the students (n = 23) were studying the fourth year of the Bachelor of Pharmacy (Honours) degree, with two students in the third year of the program. Half of the participating students had no prior experience using AR technology, 40% had limited use of 10 h or less per year, with only one student using AR technology for more than 5 h per week.

### 3.2. Effect on Student Achievement

Apart from Question 1, the differences between the pre- and post-test questions were significantly different (Table 4). The results indicated that there was a statistically significant increase in correct knowledge responses between the total pre- and post-test scores (t = 7.45, *p* < 0.001, sd = 1.42, df = 24) (Table 4).

### 3.3. Student Acceptance of AR Technology

Results for user acceptance of the AR MagicBook scored highly in all areas, as seen in Table 5. On a Likert scale of 1 (strongly disagree) to 5 (strongly agree), most students indicated a high degree of user acceptance and acceptability of the AR MagicBook, (Table 5).

### 3.4. Students’ Opinions on Using AR for Learning about Medicines

We identified recurring key words and phrases in the qualitative data that aligned with the aims of the study. Emerging themes described student experiences using the AR tool.

Theme 1 was centred on knowledge acquisition, e.g., “*It consolidated my knowledge by providing helpful videos of patient-pharmacy interaction, which greatly improved my knowledge and understanding of naloxone*”, and “*The animation/videos were interesting and made it easier for me to understand and remember the context*”.

Theme 2 was based on emotional responses and illustrated positive feelings about using the tool, e.g., “*It was interactive and engaging”, and “It was very stimulating*”, and “*The animation videos were interesting*”.

Theme 3 was related to usability of the tool, e.g., “*It was easy to follow*”.

Theme 4 related to AR MagicBook content and identified that additional content such as 3D images and subtitles could enhance the utility of the AR tool, e.g., “*It could incorporate 3D models and models that can be moved*”.

## 4. Discussion

This study was designed to develop a tool which would enable pharmacy students more flexible opportunities for engaging in learning about medicines beyond their limited formal class time, with a focus on taking a student-centred approach to learning. An interactive learning module using AR technology was developed for upskilling pharmacy students in the supply of naloxone, and presented in a MagicBook format. As this was the first time the students had experienced learning with the use of AR technology in their program this pilot study provides important information about their perceptions on its acceptability and usability as well as its effectiveness for learning. It is envisaged that future applications of this work would enable students to engage with the AR learning experience at any place and at any time throughout their pharmacy program.

In the user acceptance survey, all students agreed/strongly agreed that the AR MagicBook enabled the presentation of information on medicines as a practical patient-pharmacist interaction which enhanced and stimulated their learning, and that it could be appropriately used in a range of learning activities throughout their program. To maintain and build on these positive responses from students, it was important to consider the positive impact of AR on learning. One suggestion is that interactive technology can improve student achievement levels through an increased level of motivation [12,22,23,24]. Moreover, as the AR technology used in the current study allowed students to learn at their own pace and enabled multiple access to replay various scenarios and therefore they could reinforce difficult concepts by controlling their own learning.

Although all students agreed/strongly agreed that they thought their knowledge of medicines improved after using AR for learning, establishing the extent of these gains to determine the effectiveness of this learning strategy was important. Overall, there appeared to be a significant improvement in student knowledge about the medication naloxone after using the AR MagicBook for learning. As the results show, for four of the five test questions, there was a significant improvement between the pre- and post-test results, with an increase in the overall mean score from the pre-test (57%) to the post-test (99%), which was statistically significant at *p* < 0.001. With question one, however, most of the students (92%) obtained the correct response at baseline. This is possible given that students have prior foundation knowledge based on previous study or experience.

Similar learning outcomes were reported in a study by Albrecht et al., with a small group of 10 medical students in Germany learning about forensic medicine. However, in this study, the students were divided into an experimental group (n = 6) using AR for learning, as well as a control group (n = 4), with the experimental group showing a statistically significant gain in knowledge compared to the control (*p* = 0.03) [25]. Another study with a larger cohort of students also reported similar results for learning with AR. A group of 70 medical students in Turkey were divided into one of two groups to study anatomy, an experimental group using AR in a MagicBook, and a control group. The purpose of this study by Kucuk et al. (2016) was to determine the effects of using AR to learn anatomy by comparing the academic achievements and cognitive load of the two groups of students. The authors suggested that integrating multimedia materials into the learning process with AR enabled students to have a reduced cognitive load. Furthermore, significantly greater gains in knowledge were reported in the experiment group compared to the control (*p* < 0.05) [22]. Similar results have also been observed in other studies for learning alone [11] or in combination with increased motivation [26]. These findings are consistent with the notion that AR is starting to gain traction because of its ability to enhance content [2].

There is considerable evidence to support the observation that AR produces good learning outcomes because students who are actively involved in the learning process are better able to retain knowledge resulting in a higher level of achievement [16,25,27]. Moreover, people learn more deeply from words and pictures than words alone, a format that uses auditory and visual sensory stimulation [28]. The cognitive theory of multimedia learning suggests that learning from an organised and well-designed format with relevant resources such as videos and images can reduce cognitive effort resulting in improved student performance [26]. Xie et al. (2017) suggest that adding cues to multimedia materials reduces learners’ perceived cognitive load and significantly facilitates the retention and transfer of learning [29], and this can be successfully used as a technique to enhance learning [30]. It has been suggested that the built-in markers on AR images provide visual cues to trigger the display of virtual information. These cues are not intended to provide new information, but to give attentional guidance that encourages the user to take action, thereby reducing cognitive overload [29,30].

## 5. Limitations

A limitation of this study is the small sample size of 25 university students and the lack of a control group for the comparison of using AR for learning. However, the results gained from this pilot study provides baseline information on which to build for the use of augmented and virtual reality technologies within pharmacy education in Australia. It should also be noted that while HP Reveal^®^ was the most appropriate AR tool to use at the time of the study, with rapid changes in technology HP Reveal^®^ has now been superseded with similar software.

## 6. Conclusions

AR technology can position the learner within a real-world context whilst enabling participatory learning practices. While the results of this study require further investigation, it is clear that AR technology has the potential to engage and stimulate students in a way that could create a more inclusive learning environment. This study has identified the benefits that AR can bring to tertiary pharmacy education. AR technology can be implemented at minimal cost to the creator and the learner as the vast majority of students own mobile devices. Web-based AR platforms are easy to learn and implement and can provide educators with a simple introduction to the world of augmented and virtual reality technology. Further research is required to investigate the use of AR for learning about a range of different medications and build on the implementation of AR in tertiary pharmacy education.

## Figures and Tables

**Table 1:** Comparison of the feasibility of using Wikitude, HP Reveal^®^ and Blippar.

	Wikitude	HP Reveal	Blippar
Affordable		✓	
User Friendly		✓	✓
Ease of Design	✓	✓	✓
Available	✓	✓	✓

**Table 2:** Learning objectives for the naloxone AR MagicBook.

• Identify current trends in opioid overdoses in Australia
• Identify common signs and symptoms of an opioid overdose
• Describe how naloxone works in an opioid overdose
• Recognise precautions and contraindications for naloxone use
• Provide key counselling points to a patient obtaining naloxone from community pharmacist

**Table 3:** Pre- and post-test questions on knowledge (excluding the multiple-choice options).

Multiple choice:
1. Which of the following statements best describes the pharmacological mechanism of action of naloxone?
2. Which of the following correctly lists the symptoms of an opioid overdose?
3. Which of the following statements best describes how you would advise the patient to administer naloxone?
4. By which supply method can a patient obtain naloxone from a pharmacy in NSW?
Short answer:
5. List TWO important counselling points to provide to a patient about naloxone.

**Table 4:** Comparison of pre- and post-test results—correct responses (n = 25).

Questions	Q 1 % (n)	Q 2 % (n)	Q 3 % (n)	Q 4 % (n)	Q 5 % (n)	Mean Score % (n)
Pre-test	92 (23)	80 (20)	48 (12)	56 (14)	8 (2)	57 (71/125)
Post-test	100 (25)	100 (25)	96 (24)	100 (25)	100 (25)	99 (124/125)
∆	8 (2)	20 (5)	48 (12)	44 (11)	92 (23)	42 (53)
*p*-value	ns	0.02	<0.001	<0.001	<0.001	<0.001

**Table 5:** Results of the AR user acceptance survey (n = 25).

	Strongly Disagree	Disagree	Neutral	Agree	Strongly Agree
	%/n	%/n	%/n	%/n	%/n
The use of the AR tool stimulated my interest to learn about medicines	-	-	-	36/9	64/16
The use of the AR tool is a useful teaching resource when compared with other methods for medicines training such as lectures and tutorial exercises	-	-	12/3	32/8	56/14
The AR tool was able to present medicines information relating to a typical patient-pharmacist communication encounter	-	-	-	36/9	64/16
The AR images did not distract from identifying medicines information	-	-	12/3	48/12	40/10
I could identify improvement in my own medicines knowledge after using this AR tool	-	-	-	28/7	72/18
The AR tool could be applied to a range of learning activities in my pharmacy degree	-	-	-	20/5	80/20
